# No difference in osteoarthritis, but less graft failures after 5 years, comparing anatomic double‐bundle to anatomic single‐bundle ACL reconstruction

**DOI:** 10.1002/ksa.12528

**Published:** 2024-11-07

**Authors:** Cathrine Aga, Ingrid Trøan, Stig Heir, May Arna Risberg, Tariq Rana, Steinar Johansen, Morten Wang Fagerland, Lars Engebretsen

**Affiliations:** ^1^ Martina Hansens Hospital Gjettum Norway; ^2^ Oslo University Hospital Oslo Norway; ^3^ Oslo Sports Trauma Research Centre Oslo Norway; ^4^ Norwegian School of Sport Sciences Oslo Norway; ^5^ Lovisenberg Diaconal Hospital Oslo Norway; ^6^ Oslo Centre for Biostatistics and Epidemiology Oslo University Hospital Oslo Norway

**Keywords:** ACL reconstruction, double‐bundle, graft failure, osteoarthritis, revision

## Abstract

**Purpose:**

The purpose of this study was to compare the incidence of knee osteoarthritis (OA) between the anatomic single‐bundle (SB) and anatomic double‐bundle (DB) anterior cruciate ligament (ACL) reconstruction technique after 5‐year follow‐up (FU). Secondary objectives were to compare patient‐reported outcome measures (PROMs), clinical examination, activity level, functional tests and graft failures between the two groups.

**Methods:**

The study was a secondary analysis after 5‐year FU of a randomized controlled trial (RCT) (Clinical Trials NCT01033188). One hundred and twenty patients between 18 and 40 years were randomized to either anatomic SB or anatomic DB reconstruction. The Kellgren–Lawrence (KL) classification grade ≥2 and the Osteoarthritis Research Society International (OARSI) atlas criteria score ≥2 were used for defining OA. Additionally, PROMs were obtained and clinical examinations of the knees were performed. Finally, the number of patients experiencing graft failure in each group was recorded.

**Results:**

Radiographic imaging was performed in 39 patients in the SB group and in 37 patients in the DB group. Four patients (10%) in the SB group and two (5%) in the DB group developed osteoarthritis according to the KL classification (*p* = 0.28). Five (13%) in the SB group and three (8%) in the DB group developed osteoarthritis according to the OARSI atlas criteria (*p* = 0.59; difference 5.0% [95% confidence interval, CI: −0.10 to 0.20]). There were no significant differences in the PROMs, clinical examinations, activity levels, or functional tests when comparing the two groups. Of initially 62 SB patients, 14 (23%) experienced graft failure compared to 4 (7%) of the 58 DB patients (*p* = 0.015; difference 0.016 [95% CI: 0.03–0.29]).

**Conclusion:**

At 5‐year FU, there were no significant differences in the incidence of OA, PROMS, or other clinical findings comparing the anatomic DB to anatomic SB ACL reconstructed patients. There were fewer graft failures among patients treated with anatomic DB ACL reconstruction.

**Level of Evidence:**

Level II.

**Abbreviations**
AARSC: Anatomic Anterior cruciate ligament Reconstruction Scoring Checklist
ACL: anterior cruciate ligament
AM: anteromedial
CT: computed tomography
DB: double‐bundle
FU: follow‐up
IKDC: International Knee Documentation Committee
JSN: joint space narrowing
KL: Kellgren–Lawrence
KOOS: Knee Injury and Osteoarthritis Outcome Score
MRI: magnetic resonance imaging
OA: osteoarthritis
OARSI: Osteoarthritis Research Society International
PL: posterolateral
PROM: patient‐reported outcome measure
PTOA: post‐traumatic osteoarthritis
RCT: randomized controlled trial
ROM: range of motion
SB: single‐bundle
SPSS: Statistical Package for Social Sciences

## BACKGROUND

The anterior cruciate ligament (ACL) rupture is one of the most common ligament injuries occurring in the young and active population. More than 130,000 reconstructions are now performed yearly in the United States [28]. Only 60% of the ACL reconstructed patients are able to return to the same level of activity and ACL‐injured patients are at high risk of developing post‐traumatic osteoarthritis (PTOA) [4, 35]. The anatomic double‐bundle (DB) reconstruction technique of the ACL has been shown to normalize the in situ forces and improve anterior and rotational stability of the knee compared to traditional non‐anatomic single‐bundle (SB) ACL reconstruction [12, 31, 40, 54, 55]. The DB ACL concept considers the ACL to consist of two individual bundles anatomically and kinematically; the posterolateral (PL) and anteromedial bundle (AM) [33, 38] [20]. Simultaneously with the introduction of the DB concept, the anatomic ACL reconstruction technique has gradually been implemented [29] [49, 50]. Transtibial drilling and offset guides have been replaced by anteromedial (AM) portal drilling of the femoral tunnel and placement according to anatomic landmarks. Despite initial promising findings, short‐term clinical studies have found minimal beneficial effects on patient‐reported outcome measures (PROMs) and other clinical outcomes comparing anatomic DB to anatomic SB reconstruction technique [2, 17, 39]. Small differences in rotatory and sagittal laxity in favour of the anatomic DB reconstruction were found, but the quality of life and the clinical outcome for the patients were equal compared to an anatomic SB ACL reconstruction [24, 30]. The anatomic DB ACL reconstruction procedure has been found expensive, time‐consuming, and surgically demanding with questionable short‐term clinical benefit compared to the anatomic SB reconstruction [8, 16].

Graft failure and revision surgery have a high impact on the affected patients. The anatomic DB ACL reconstruction technique has normal in situ forces on the graft, and the grafts achieve a thicker total graft size compared to anatomic SB reconstructions. This could possibly reduce the risk of graft failure. Clinical studies comparing DB to SB ACL reconstruction with mid‐term FU have so far been few and with conflicting results [11, 24]. There is a need for high‐quality studies with longer FU to determine whether anatomic DB reconstructions could still be an option for ACL‐injured patients [23].

PTOA is a known complication after ACL injury. In a recent study, PTOA was detected in 1.7% of ACL reconstructed patients after 2 years and in 13.6% of the patients after 10 years [35]. The PTOA seems to evolve despite surgical intervention after ACL injury, but altered loading about the injured knee seems to be a contributing factor [32, 47, 51, 56]. Anatomic SB ACL reconstruction counteracts the degenerative cartilage changes in the knee compared to non‐anatomic reconstructions [37]. Additionally, anatomic DB reconstructed knees produce less lateral antero‐posterior and rotatory laxity compared to the anatomic SB reconstruction in a laboratory setting [34]. However, it is still uncertain whether these kinematic improvements could affect the clinical outcome and reduce the risk of OA development in the long term [37, 39].

The aim of this follow‐up (FU) of a prospective randomized controlled study was to compare anatomic DB versus anatomic SB ACL reconstruction technique more than 5 years after surgery regarding the development of osteoarthritic changes. Furthermore, to compare PROMs, clinical outcomes, and the incidence of graft failures between the two techniques. Our hypothesis was that the anatomic DB ACL reconstructed patients would have a lower incidence of osteoarthritic changes as judged by the Kellgren–Lawrence (KL) classification system of the affected knees compared to anatomic SB ACL reconstructed patients at 5‐year FU after surgery.

## METHODS

The study is an FU of a prospective randomized controlled trial (RCT) (ClinicalTrials.gov identifier: NCT01033188) looking at two different surgical reconstruction techniques of ACL reconstruction with hamstring tendon grafts with 2‐year FU as the primary endpoint [2]. The study was approved by the Regional Committee for Medical and Health Research Ethics, South‐Eastern Norway. Study participants were recruited at two different hospitals (Oslo University Hospital and Martina Hansens Hospital) during the inclusion period. The inclusion period was from 1 January 2010 to 18 June 2015. Before inclusion, all patients signed a written consent form. The patients were followed at baseline and at 1, 2 and 5 years after surgery, and all examinations were performed by the three study assessors (IT, CA and LE). The initial study included 120 patients with symptoms from the knee due to a primary ACL injury [2]. The patients were 18–40 years old and had completed a minimum of 2 months of knee‐specific rehabilitation supervised by a physical therapist. If the patients still had symptoms from their ACL injury after rehabilitation, they were asked to participate in the current study. Patients with a contralateral or partial ACL injury; injury to the posterior cruciate ligament, PL corner, or lateral collateral ligament; and medial collateral ligament injury with residual medial instability of the knee were excluded. Patients with established OA (KL ≥ 2), large meniscal resections (more than 50% of the menisci), and insufficient hamstring tendon graft size (<5 mm for PL and <6 mm for AM bundle) at surgery were also excluded.

### Intervention

**Figure 1:**
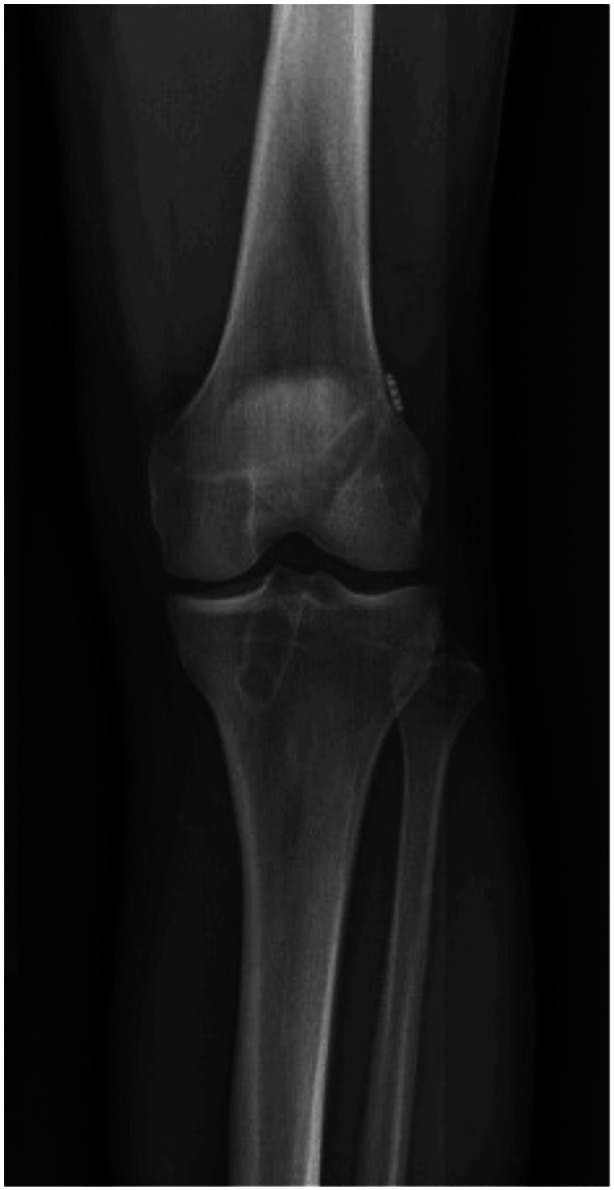
X‐ray image at 5 years of a single‐bundle ACL reconstructed knee. ACL, anterior cruciate ligament.

**Figure 2:**
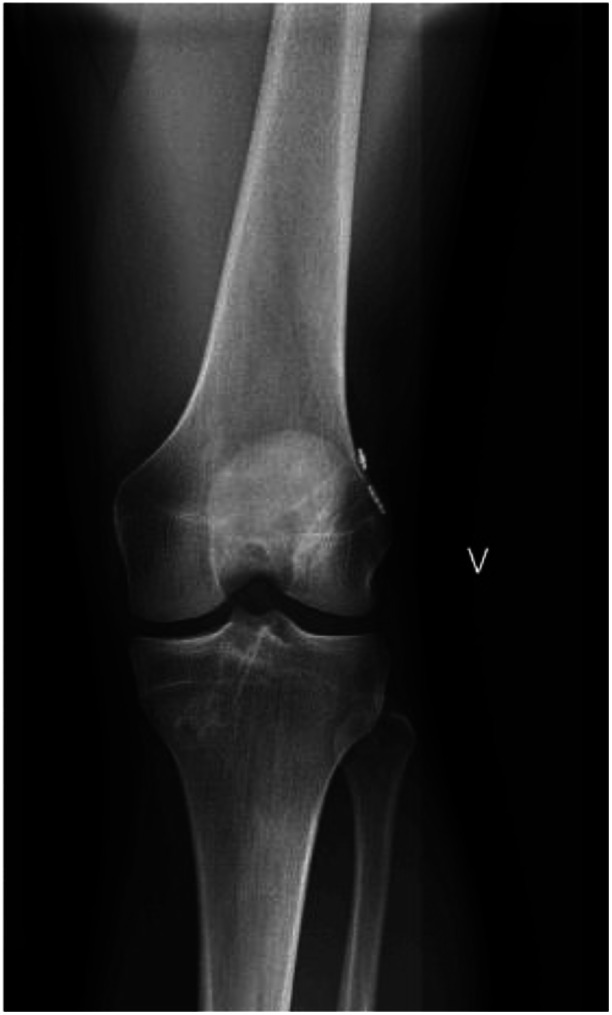
X‐ray image at 5 years of a double‐bundle ACL reconstructed knee. ACL, anterior cruciate ligament.

Anatomic ACL reconstruction with hamstring tendon autografts was performed either with SB or DB reconstruction technique (Figures 1 and 2). One single surgeon (S. J.) performed all the surgical procedures except for two of the patients. The surgeon did not assist or participate in any of the patients' 1‐, 2‐ and 5‐year assessments.

### Anatomic SB ACL reconstruction

An accessory AM portal was used for the establishment of the femoral tunnel; then a Steadman awl was positioned at the centre of the femoral footprint and drilled according to the anatomic landmarks with the same size as the measured graft size (Figure 1). The centre of the tibial footprint was identified according to anatomic landmarks [57]. With an external tibial guide, the tibial tunnel was drilled at the same size as the measured graft size. The graft was passed through the tunnels, and finally, fixation was performed on the femoral side with a suspension device (Endobutton CL; Smith & Nephew). Then the graft was cycled through 20 flexion‐extension movements before fixation on the tibial side at 20° of flexion with a PEEK interference screw (Biosure PK; Smith & Nephew). The average graft size for the SB reconstructions was 8 mm in diameter for the femoral tunnel and 9 mm for the tibial tunnel.

### Anatomic DB ACL reconstruction

An accessory AM portal was used to establish the femoral tunnel (Figure 2). First, the centre of the AM bundle footprint was marked according to anatomic landmarks, and the femoral AM tunnel was drilled to the same size as the measured graft size [57]. For the PL bundle, a DB femoral drill guide (Anatomic ACL‐PL Femoral Aimer; Smith & Nephew) was used. On the tibial side, an external tibial guide was used. First, the AM tunnel was drilled, then the PL Tibial Aimer guide (Anatomic ACLPL Tibial Aimer; Smith & Nephew) was placed in the AM tunnel according to the anatomic landmarks and the PL tunnel was drilled. The tunnels were drilled in the size of the measured graft size. The grafts were passed through the tunnels, and graft fixation on the femoral side was carried out with two suspension devices (Endobutton CL; Smith & Nephew). The grafts were then cycled through 20 flexion‐extension movements before fixations were performed on the tibial side with two PEEK interference screws (Biosure PK; Smith & Nephew) at 60° of flexion for the AM bundle and at full extension for the PL bundle. The average graft sizes were 7 mm in diameter for femoral and tibial side of the AM bundle and 6 mm in diameter for the femoral and tibial side of the PL bundle.

### Rehabilitation

Mobilization on crutches with weight bearing was recommended from the first postoperative day. If the menisci were sutured, partial weight‐bearing and flexion beyond 90° were restricted for the first 6 weeks. The patients were advised to continue to undergo strength and neuromuscular training guided by a physical therapist during the first 9 months after surgery and to avoid pivoting sports during the same period.

## OUTCOMES

### Radiographic assessments

The main objective of the study was to compare the incidence of osteoarthritic changes in the anatomic DB compared to the anatomic SB ACL reconstructed patients 5 years after surgery, as judged by the KL classification. The radiographic imaging was performed as standing anterior–posterior (AP) radiograph of both legs in a Synaflexer™ X‐ray positioning frame (Synarc Inc) [10]. The x‐rays were judged by an independent trained musculoskeletal radiologist. The KL grading system of osteoarthritis in the joint was used for classification [26]. OA was defined as KL grade 2 or more (grades 2, 3 and 4) on anterior‐posterior standing radiographs of the affected and unaffected knee. Further objectives were to compare the same radiographic imaging between the two groups with the ‘Osteoarthritis Research Society International (OARSI) grading system’ [3]. OARSI criteria have been established as a semiquantitative separate scoring system for osteoarthritis of the joints, including osteophytes and joint space narrowing (JSN) for each compartment (medial and lateral) of the knee. If the sum of JSN and osteophytes from the medial and lateral compartment was grade 2 or more, or if grade 1 JSN occurred together with grade 1 osteophytes, tibiofemoral OA was defined.

### Patient‐reported outcome measures

Additional secondary objectives were to compare the five KOOS subscales (Symptoms, Pain, Activities of Daily Living, Quality of Life and Sports/Recreation) and the International Knee Documentation Committee (IKDC) 2000 subjective knee evaluation form between the two groups [21, 36]. The sports participation and activity level were documented by the Tegner activity score, the Activity scale, and the return to sports rate between the two groups [6, 46]. Return to sports was defined as returning to the same main sport as before the injury at 5‐year FU.

### Clinical assessments and functional tests

A clinical examination was performed on the affected and unaffected knees. Anteroposterior laxity was assessed by the Lachman's test and graded in 0 (−2mm) + 1 (3–5 mm), +2 (6–10 mm) or +3 (>10 mm) compared with the uninvolved knee [21, 48]. The anteroposterior laxity was also measured by the KT 1000 arthrometer (MEDmetric); the translation was compared with the uninvolved knee at 134 N and at MM displacement [15]. Rotatory laxity was subjectively assessed by the pivot shift tests [41]. Pivot shift was assessed by the Slocums test and graded from 0 to +3. Functional performance of the knee was measured by the single‐legged hop test comparing the hop distance of the unaffected with the affected knee and presented as the difference in cm between them [27].

### Graft failures

Finally, all patients were questioned as to whether they had experienced any knee‐specific adverse events or reoperations after reconstruction. The graft ruptures and ACL revision surgeries were assessed for each group. Details from these events were obtained from the medical journal. A graft rupture was defined if there was a total rupture of the ACL reconstruction, defined by clinical examination (Lachman ≥2+ and/or pivot‐shift test positive) and shown on magnetic resonance imaging (MRI) or at second‐look arthroscopy. Revision reconstruction was defined as a reoperation with the reconstruction of a new ACL graft in the same knee.

### Sample size calculation, randomization and blinding

The details of the sample size calculation have been published previously [2]. The process of randomization and deviations from the study protocol have also been described previously. The outcome assessors, radiologists and patients were not blinded at the 5‐year FU, but the statistical advisor was blinded for the intervention when performing the analysis.

### Statistical analysis

The statistical analysis was made according to the statistical analysis plan presented in Clinical Trials (ClinicalTrials.gov identifier: NCT01033188). All continuous variables were summarized with means and standard deviations (SDs) within each treatment. Categorical data were summarized with counts and percentages within each category and treatment arm. The PROMs were analysed with a linear mixed model, which included fixed effects for treatment, time point (baseline, 1 year, 2 years and 5 years), and treatment × time point interaction as well as a random intercept. Between‐treatment differences were presented in values at 5 years with 95% confidence interval (CI). The remaining continuous variables (KT 1000 and one‐leg hop test) were analysed with the two‐sample *t* test. Categorical outcomes were analysed with the Mann–Whitney *U* test and dichotomous outcomes were analysed with the Newcombe hybrid score CI and the Fisher mid *P* test [19]. The significance level was set at *p* < 0.05. Stata/SE 18.0 (StataCorp LLC) was used to perform the statistical analyses.

## RESULTS

**Figure 3:**
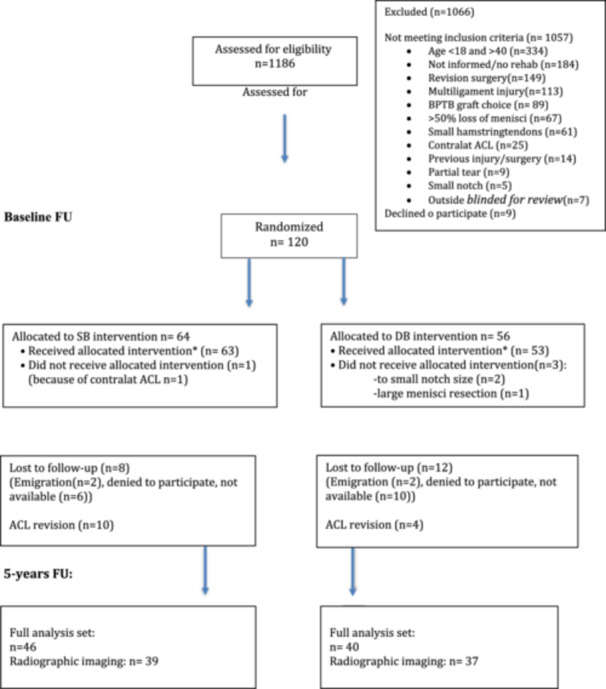
Flow chart. The figure presents the number of patients allocated, randomized, and the number of patients assessed at baseline and at 5‐year follow‐up. DB, double‐bundle; SB, single‐bundle.

Of the initial 120 included patients, fourteen patients were excluded from clinical examination at 5‐year FU because their graft had been revised and substituted by a new graft in the affected knee. Of the remaining 106 patients, 46 patients in the SB and 40 patients in the DB group completed the 5‐year clinical FU (Figure 3). Patient demographic and surgical details at Baseline are listed in Table 1.

**Table 1:** Patient demographic and surgical details at baseline.

	Age at surgery (years) (mean ± SD)	Male sex, *n* (%)	BMI at surgery (mean ± SD)	Tegner preinjury (mean ± SD)	Time from injury to surgery (months) (mean ± SD)	Meniscal injuries, *n* (%)	Chondral lesions, *n* (%)
DB group (56)	27.4 ± 6.3	47 (84)	25.1 ± 2.9	7.9 ± 1.2	15.5 ± 18.2	26 (46)	10 (18)
SB group (64)	27.1 ± 5.5	41 (64)	24.5 ± 3.1	7.7 ± 1.5	15.7 ± 20.3	33 (52)	13 (20)

*Note*: The values are given in mean ± standard deviation (mean ± SD) or in percentages (%).

Abbreviations: BMI, body mass index; DB, double‐bundle; SB, single‐bundle; SD, standard deviation.

### Radiographic assessments

**Table 2:** Radiographic assessments of the affected knees.

Kellgren–Lawrence (KL) score	SB (%), *n* = 39	DB (%), *n* = 37	*p* *
Grade 0	34 (87)	35 (95)	0.28
Grade 1	1 (3)	0	
Grade 2	4 (10)	2 (5)	
Grade 3	0	0	
Grade 4	0	0	
OARSI OA^a^	5 (13)	3 (8)	0.59 (−0.10 to 0.20)

*Note*: OA changes assessed by the KL score.

Abbreviations: CI, confidence interval; DB, double‐bundle; OA, osteoarthritis; OARSI, Osteoarthritis Research Society International; SB, single‐bundle.

^a^ OARSI defined OA in each group.

* *p* Value for between‐group difference probabilities (95% CI).

According to the KL classification score, the current study showed an overall development of OA of 8%.

Four (10%) patients in the SB group and two (5%) in the DB group developed OA according to the KL classification at 5‐year FU (*p* = 0.28). Five (13%) in the SB group and three (8%) in the DB group developed osteoarthritis according to the OARSI atlas criteria (*p* = 0.59; [95% CI: −0.10 to 0.20]) (Table 2). In the contralateral knee, two patients (3%) developed OA, both patients had SB reconstruction and showed bilateral degenerative changes.

### PROMs, clinical assessments and functional tests

**Table 3a:** Results PROMs.

	SB group, *N* = 46 (95% CI)	DB group, *N* = 40 (95% CI)	*p* *
IKDC 2000 subj score	69 (66–73)	72 (68–76)	0.45
KOOS Pain	91 (87–95)	93 (88–97)	0.56
KOOS Symptoms	89 (85–94)	91 (87–96)	0.53
KOOS ADL	96 (92–99)	97 (93–101)	0.56
KOOS Sport/Rec	77 (71–84)	81 (75–88)	0.40
KOOS QoL	72 (66–77)	76 (70–82)	0.33

*Note*: Data are shown as mean value (95% CI) at 5‐year FU.

Abbreviations: CI, confidence interval; DB, double‐bundle; FU, follow‐up; IKDC, International Knee Documentation Committee; KOOS, Knee Injury and Osteoarthritis Outcome Score; PROM, patient‐reported outcome measure; SB, single‐bundle.

* *p* Value of between‐group mean differences.

**Table 3b:** Results activity level and functional test.

	SB group	DB group	*p* *
One‐leg hop test (cm) (95% CI)	4.1 (−1.2 to 9.3)	1.5 (−5.5 to 8.5)	0.55
Return to previous main sports (*n*) yes/no	9/14	7/10	0.87
Tegner score (*n*) 1–10	0/0/10/2/12/10/8/1/2	0/2/2/6/9/5/11/0/5	0.39
Activity scale (*n*) 1–4	17/28/0/0	12/23/2/3	0.39

*Note*: Data are shown as number (*n*) or mean value (95% CI).

Abbreviations: CI, confidence interval; DB, double‐bundle; SB, single‐bundle.

* *p* Value of between‐group difference at 5‐year follow‐up.

**Table 3c:** Results of clinical assessment.

	SB group, *N* = 46 (95% CI)	DB group, *N* = 40 (95% CI)	*p* *
Lachman's test			0.25
0	15	16	
1	27	24	
2	4	0	
3	0	0	
Pivot shift test			0.13
0	35	35	
1	9	3	
2	2	1	
3	0	0	
KT1000 manual maximum	2.2 (1.5–2.9)	2.0 (1.2–2.8)	0.68
KT 1000 134 N	1.6 (1.1–2.2)	1.7 (1.0–2.3)	0.96

*Note*: Data are shown as numbers (*n*) or mean value (95% CI) at 5‐year FU.

Abbreviation: CI, confidence interval; DB, double‐bundle; FU, follow‐up; SB, single‐bundle.

* *p* Value of between‐group mean differences.

The PROMs revealed no further differences between the two groups in the KOOS Pain (*p* = 0.56), KOOS Symptoms (*p* = 0.53), KOOS ADL (*p* = 0.56), KOOS Sport (*p* = 0.40) and KOOS QoL (*p* = 0.33) subscores or in the IKDC 2000 subjective score (*p* = 0.45) (Table 3a). The activity level assessed by the Tegner score, Activity scale and return‐to‐sports rates revealed no further differences between the two groups (Table 3b).

There were no significant differences in clinical laxity measures including Lachman's test (*p* = 0.25), pivot shift test (*p* = 0.13), and the KT 1000 at manual maximum (MM) (p = 0.68) and at 134 N (*p* = 0.96) between the two groups (Table 3c). One‐leg hop test revealed no further difference between the two groups (*p* = 0.55) (Table 3c).

### Graft failures

**Figure 4:**
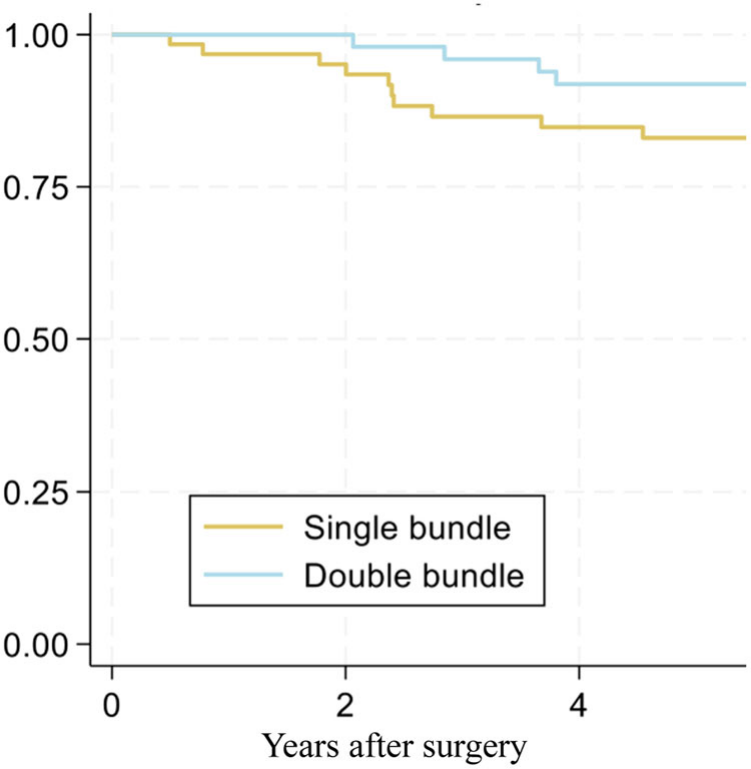
The Kaplan–Meier plot shows the estimated survival without revision surgery for anatomic SB (yellow) and anatomic DB (blue) ACL reconstructions. ACL, anterior cruciate ligament; DB, double‐bundle; SB, single‐bundle.

There was a significant difference between the two groups when it came to graft failures. Fourteen (23%) patients experienced a graft failure in the SB group compared to 4 (7%) in the DB group (*p* = 0.015; [95% CI: 0.03%–0.29%]). Revision surgery was performed in 10 (16%) out of 62 SB versus 4 (7%) out of 58 DB (*p* = 0.089; [95% CI: −0.04 to 0.20]) (Figure 4).

## DISCUSSION

### Radiographic outcomes

In this FU of a randomized controlled study comparing anatomic DB reconstruction with anatomic SB reconstruction technique after 5 years, no difference in the incidence of knee OA was found. In the DB group, 5% of the patients expressed osteoarthritic changes in their affected knee, while 10% of the SB reconstructions had osteoarthritic changes after 5 years. OA incidence was lower than detected in other studies following anatomic ACL reconstructed patients: In one study with mid‐term FU, the incidence of OA in anatomic SB and anatomic DB reconstructed knees was 20% in the medial compartment and 10% in the lateral compartment after 5‐year FU. The study found no difference in OA development between the two techniques [43]. The relatively higher amount of OA changes in their study could be explained by the older age of the study participants and a higher amount of patients with meniscal resections compared to our study. In another study with more than 10‐year FU of 31 anatomic SB and 37 anatomic DB patients, they also revealed no difference in OA changes between the two techniques using three different scoring systems for OA (KL, Ahlbäck and the Fairbank classification system) [5].

In the current study, there were two patients with OA in the contralateral knee. They both had an SB ACL reconstruction, and both showed OA development in the affected and in the non‐affected knee. Alteration of kinematics because of an ACL injury could possibly affect the development of OA also in the contralateral knee together with underlying genetic and morphologic risk factors for OA development [7].

### PROMs and clinical outcomes

In accordance with previous studies, this study found no difference in the clinical outcomes between the two techniques for PROMS, clinical assessments, activity level and functional scores [5]. Recently, a systematic review evaluated RCTs with more than 5‐year FU. The review concluded that there was no difference between the DB and SB surgical techniques regarding the development of osteoarthritis, clinical outcomes, and graft failures [11]. However, some of the included studies have used non‐anatomic reconstruction techniques and some included other grafts than hamstring tendon grafts for the SB ACL reconstruction. In the last decade, there has been a change towards an anatomic reconstruction technique in ACL‐reconstructed patients. Some of the reviews comparing the two techniques have compared non‐anatomic or quasi‐anatomic procedures. In the current study, the anatomic DB and SB reconstructions were aimed at all the patients, and the tunnel placement was verified by postoperative 3D CT scans of the knees [2].

This study confirmed that the quality of life and activity level were not improved by the anatomic DB procedure. Many authors now conclude that there is no use in performing the anatomic DB procedure in an unselected patient group, considering the fact that this procedure is both more cost‐ and time‐consuming and has little or no benefit in clinical outcomes compared to the anatomic SB reconstruction procedure [5].

### Graft failure outcome

The current study revealed a significantly higher amount of graft failures in the anatomic SB compared to the anatomic DB group. Järvelä et al. found that 10 out of 60 anatomic SB reconstructed patients were diagnosed with a graft failure at 10‐year FU compared to only 1 out of 30 in the anatomic DB group [24]. The difference was explained by the learning curve for the anatomic SB procedure and by the excessive micromotion in knees with SB reconstructions compared to DB reconstructions. It was assumed that anatomic DB reconstructions had some kinematic superiority that could prevent a new trauma and that the thicker grafts and a double fixation of the grafts in the DB group could lead to fewer graft ruptures. In a meta‐analysis comparing DB to SB reconstructed patients, the “The anatomic ACL reconstruction scoring Checklist” (AARSC) was used to screen for the eligibility of the anatomic reconstruction technique. The study included 13 RCTs with patients reconstructed with strictly anatomic reconstruction technique, and they detected an overall higher complication rate for anatomic SB versus anatomic DB reconstructed patients [53]. In a review comparing clinical studies with more than 20‐year FU, graft failures were found in 2%–18.5% of the patients [18]. Younger age, preoperative laxity, and chronic ACL tear together with medial meniscal resection and increased lateral tibial slope are all risk factors for abnormal laxity post‐operatively [13]. The current study was not designed to define whether these risk factors could explain the high graft failure rate, but a further analysis of this subgroup is planned.

The morphology of the ACL could influence the risk of primary ACL rupture. Decreased cross‐sectional area of the ACL is a known risk factor for primary ACL rupture [52]. DB reconstructed knees have a higher total graft size than SB reconstructions, and in a case–control study from the Swedish ligament register, Snaebjørnsson et al. confirm that increased graft diameter reduces the risk of revision surgery [9, 42, 44]. In another registered study comparing DB and SB reconstructions in Scandinavia, it was found that SB reconstructions had an increased risk of revision surgery compared to DB reconstructions [1, 45]. The national register studies only detect revision surgery, and this does not necessarily reflect the real amount of graft failures. Particularly in DB reconstructed patients, the surgeons could be reluctant to perform revision surgery because of more extensive bone loss at the tunnel sites and the increased need for two‐staged revision surgery [25].

Four patients in the current study with graft failure were still not revised. This was because they did not need revision surgery or were waiting for revision surgery. There was a gender difference between the two groups.

At baseline, the DB group consisted of only 18% women, and the SB group had 34% women. This could affect the revision outcome, but even though female gender is a known risk factor for primary ACL rupture, this has not been shown for revision surgery [22].

## LIMITATIONS

The study was performed by a single surgeon at two different hospitals with a high volume of these elective procedures. The results from this study are therefore not necessarily applicable to outcomes for all patients going through ACLR. In the current study, 20 patients were lost to FU and 14 patients with revision reconstruction were also excluded from the radiographic and clinical analysis because their grafts had been replaced with bone‐patella‐tendon‐bone grafts. A higher FU rate could have given strength to the study. Also, the power calculation was based on the primary outcome of the 2‐year FU, and new calculations were not performed for the secondary outcomes presented. Although radiography has a limited ability to detect OA at very early stages, the OARSI atlas criteria have been used to improve the detection at an early stage [14]. In the current study, there were only minor differences between the two grading systems. MRI scans for the detection of early OA changes could also have improved the study. To fulfil the intention‐to‐treat principle, a subanalysis looking at OA development in all randomized patients and also the patients that had performed revision‐reconstruction was performed (Table 4). This subanalysis did not reveal any further difference in the incidence of OA outcomes between the two groups. Finally, post‐traumatic OA is a slow, degenerating process, and the appearance of osteoarthritic changes is relatively late perceptible. Radiographic outcomes at 10 years or longer after surgery would have been preferable to draw conclusions about the OA incidence between the two techniques.

**Table 4:** Radiographic assessments of the affected knee including revision ACL.

Kellgren–Lawrence (KL) score	SB (%), *n* = 46	DB (%), *n* = 40	*p* *
Grade 0	40 (87)	37 (92)	0.39
Garde 1	1 (2)	1(3)	
Grade 2	5 (11)	2 (5)	
Grade 3	0	0	
Grade 4	0	0	
OARSI OA^a^	6 (13)	4 (10)	0.62 (−0.11 to 0.17)

*Note*: OA changes assessed by the KL score.

Abbreviations: ACL, anterior cruciate ligament; CI, confidence interval; DB, double‐bundle; OA, osteoarthritis; OARSI, Osteoarthritis Research Society International; SB, single‐bundle.

^a^ OARSI defined OA in each group.

* *p* Value for between‐group difference probabilities (95% CI).

This study contains a mid‐term FU of a relatively large cohort of anatomic SB and anatomic DB reconstructed patients. The anatomic placement of the tunnels was verified by 3D CT [2]. The OA was assessed both with the KL score and the more sensitive OARSI classification system. The study found a lower graft failure rate in the anatomic DB ACL reconstructed group, and it could be debated whether this procedure should be preferred for a selected group of patients. The ACL injury usually affects the younger, and active population and a DB procedure includes relatively more bone loss. The operation itself is more complicated and revisions are more challenging than in SB reconstructions. It should therefore not be recommended as a first‐line treatment and other options to prevent graft re‐ruptures should be considered instead, including graft‐type, ‐fixation, addressing additional injuries and considering extraarticular procedures [13].

## CONCLUSION

In conclusion, the current study did not reveal any difference in the incidence of OA changes, PROMs, laxity measures or activity levels between anatomic DB and anatomic SB ACL reconstruction 5 years after surgical treatment. However, the study revealed an increased risk of graft failures in the anatomic SB group compared to the anatomic DB group. The anatomic DB reconstruction does not reduce the risk of OA at 5 years but could appear protective against graft failures in ACL‐reconstructed patients.

## AUTHOR CONTRIBUTIONS

All authors have contributed substantially to the conception, design, analysis and interpretation of the data in this manuscript and will take responsibility for the content.

## CONFLICT OF INTEREST STATEMENT

Ingrid Trøan received funding from Smith + Nephew, and Lars Engebretsen has been a consultant for Smith + Nephew and Arthrex. The other authors declare no conflict of interest.

## ETHICS STATEMENT

Ethical approval was obtained from the Regional Committee for Medical and Health Research Ethics, south‐eastern Norway. A written, informed consent was received from all included patients.

## Data Availability

All data are saved at the research server from Oslo University Hospital and accessible to the authors.
